# Does the Health Insurance Program Reduce Health-Induced Poverty?: New Evidence From the China Household Income Project

**DOI:** 10.1093/geroni/igab046.1373

**Published:** 2021-12-17

**Authors:** Yalu Zhang, Qin Gao

**Affiliations:** 1 Peking University, Beijing, Beijing, China (People's Republic); 2 Columbia University, New York, New York, United States

## Abstract

The growing cost of healthcare services has been a concern for many countries in the world. In China, medical expenditures can account for as much as 65% of per capita income in some low-GDP counties in 2011. One of the primary goals of the New Rural Cooperative Medical Insurance (NRCMI) is to provide financial protection and alleviate the financial burdens of rural residents in China. This paper examined whether NRCMI participation impacted the incidence of catastrophic health expenditure (CHE) among middle-aged and older adults (45 years old and above) using the China Household Income Project 2007 rural data and an instrumental variable estimation method in two provinces where there was heterogeneity in NRCMI implementation schedule. The results show that NRCMI enrollment could not impact the likelihood of experiencing CHE among middle-aged and older adults. However, NRCMI participation increased the actual amount of medical expenses in one province but not in the other. Although none of the prior studies have used instruments and village fixed effects or take endogeneity issues into account to investigate the impact of NRCMI on relative financial burden among recipients, the results found in this study are generally aligned with the prior findings, especially with those using quasi-experimental studies. Findings from this study provide empirical evidence to the policymakers that the effect of NRCMI participation on financial protections is limited despite its broad population coverage. The limited effects are probably due to the low reimbursement rate and more utilization of expensive healthcare services.

